# Ferritinophagy: research advance and clinical significance in cancers

**DOI:** 10.1038/s41420-023-01753-y

**Published:** 2023-12-18

**Authors:** Jiewen Wang, Nayiyuan Wu, Mingjing Peng, Linda Oyang, Xianjie Jiang, Qiu Peng, Yujuan Zhou, Zuping He, Qianjin Liao

**Affiliations:** 1 Key Laboratory of Model Animals and Stem Cell Biology in Hunan Province, School of Medicine, Hunan Normal University, 371 Tongzipo Road, Changsha, 410013 China; 2 Engineering Research Center of Reproduction and Translational Medicine of Hunan Province, Changsha, 410013 China; 3 Hunan Key Laboratory of Cancer Metabolism, Hunan Cancer Hospital and the Affiliated Cancer Hospital of Xiangya School of Medicine, Central South University, Changsha, 410013 Hunan China

**Keywords:** Cancer metabolism, Cancer therapy

## Abstract

Ferritinophagy, a process involving selective autophagy of ferritin facilitated by nuclear receptor coactivator 4 (NCOA4), entails the recognition of ferritin by NCOA4 and subsequent delivery to the autophagosome. Within the autophagosome, ferritin undergoes degradation, leading to the release of iron in the lysosome. It is worth noting that excessive iron levels can trigger cell death. Recent evidence has elucidated the significant roles played by ferritinophagy and ferroptosis in regulation the initiation and progression of cancer. Given the crucial role of ferritinophagy in tumor biology, it may serve as a potential target for future anti-tumor therapeutic interventions. In this study, we have provided the distinctive features of ferritinophagy and its distinctions from ferroptosis. Moreover, we have briefly examined the fundamental regulatory mechanisms of ferritinophagy, encompassing the involvement of the specific receptor NCOA4, the Nrf2/HO-1 signaling and other pathways. Subsequently, we have synthesized the current understanding of the impact of ferritinophagy on cancer progression and its potential therapeutic applications, with a particular emphasis on the utilization of chemotherapy, nanomaterials, and immunotherapy to target the ferritinophagy pathway for anti-tumor purposes.

## Facts

- Ferritinophagy plays a crucial role in the maintenance of intracellular iron homeostasis.
- Ferritinophagy is involved in the occurrence and development of cancer.
- Ferritinophagy may be a target for anti-cancer intervention in the future.

## Open questions

- What are the pathways regulating ferritinophagy in different cancers?
- How to exert anti-tumor effects by combining ferritinophagy with cancer immunity or drugs?
- How does ferritinophagy apply to the clinic?

## Introduction

Ferritinophagy is a selective form of autophagy that specifically targets intracellular ferritin for degradation. Ferritin is a protein complex composed of ferritin heavy chain (FTH1) and ferritin light chain (FTL) subunits, which functions to bind and store excess iron within cells [1]. Ferritinophagy plays a crucial role in maintaining intracellular iron homeostasis by facilitating the degradation and recycling of stored iron, thereby enabling its utilization in cellular processes, while concurrently mitigating iron-induced oxidative damage resulting from excessive iron accumulation [2]. Mancias et al. first identified and named the process of ferritinophagy by a discovering nuclear receptor coactivator 4 (NCOA4), a cargo receptor for iron autophagic degradation, through quantitative proteomics, and its mediated ferritin turnover contributes to elevated intracellular iron levels and ferroptosis [3]. The process of ferritinophagy is initiated under conditions when the levels of intracellular iron become low. When iron levels are low, the nuclear receptor corepressor of RE1-silencing transcription factor binds to the ferritin promoter region, leading to the transcriptional activation of the ferritin heavy and light chains [4], which resulting in an increasing of ferritin level and formation of ferritin nanoparticles within the cell. When iron levels rise again, autophagy receptors such as NCOA4 bind to ferritin nanoparticles and target them for degradation through the autophagy-lysosome pathway. The process of ferritinophagy ultimately leads to the release of iron ions from degraded ferritin molecules into the cytoplasm, allowing them to be utilized for cellular functions such as heme synthesis or the generation of ROS [5]. Ferritinophagy has been shown to play an important role in a variety of physiological processes, including cellular differentiation, erythropoiesis, and immune response [2]. Furthermore, dysregulation of ferritinophagy has been implicated in the pathophysiology of a variety of diseases, including cancer [6], neurodegenerative diseases [7], and iron overload disorders such as hemochromatosis [8]. Therefore, understanding the potential mechanisms of ferritinophagy may provide effective therapeutic strategies for the treatment of cancer. In this review, we summarize the recent progresses in ferritinophagy research and discuss the applications of ferritinophagy in cancer therapy.

## Main text

### Characteristics of ferritinophagy

#### Ferritinophagy and autophagy

**Fig. 1:**
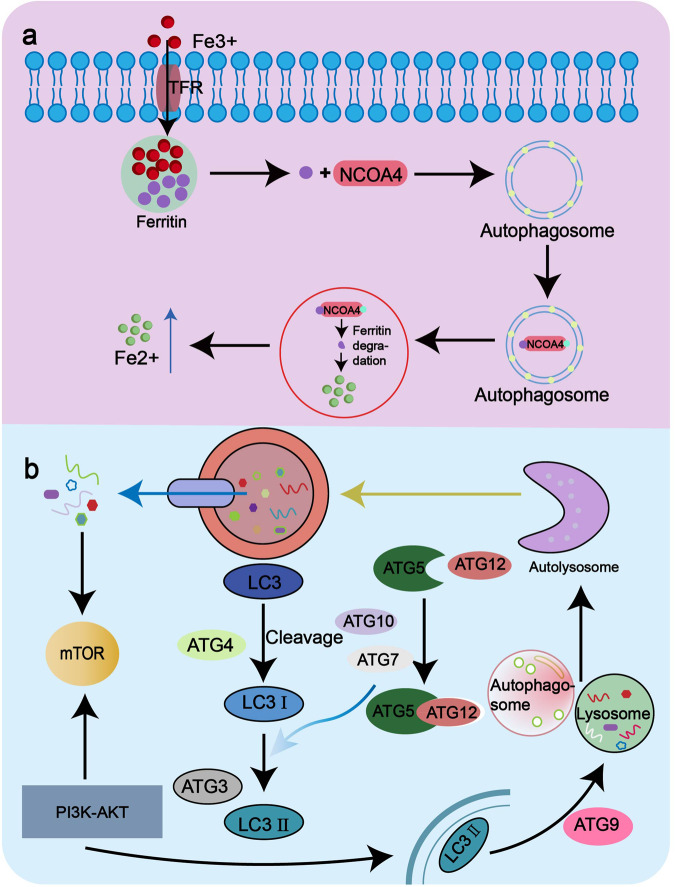
Autophagy pathway and ferritinophagy pathway. **a** Ferritinophagy pathway. Transferrin receptor 1 (TfR1) interacts with Fe to transport iron into the cell where it is stored in the form of a ferritin complex. Subsequently, NCOA4 binds directly to FTH1 of the ferritin complex and is transported to the autophagosome. It binds to LC3 and is then transported to the autolysosome, where the ferritin complex is degraded to release ferrous. **b** Autophagy pathway. The substances to be degraded in the cell are first encapsulated by autophagy vesicles and then degraded by lysosomes. In the process of autophagy formation, cytoplasmic LC3-I generates lipidized LC3-II through the participation of ATG7 and ATG3. It attaches to the autophagosome membrane to form autophagosome membrane type LC3-II. LC3-II in the membrane was degraded by lysosomes along with the encapsulated contents.

Ferritinophagy is a process by which cells degrade and recycle ferritin. And this process is mediated by autophagy, a cellular process in which damaged or unwanted cellular components are engulfed by double-membrane structures called autophagosomes and targeted for degradation by lysosomes. Ferritinophagy is a selective form of autophagy, and excess intracellular ferritin can induce iron-mediated apoptosis through autophagy by the selective autophagy receptor NCOA4 [9] which is the special molecule distinguished from other forms of autophagy (Fig. 1a). Furthermore, the process of ferritinophagy-induced cell death leads to the release of the proteoglycan decorin. This release serves as a signaling mechanism to activate both innate and adaptive immune responses, as well as the release of inflammatory factors including tumor necrosis factor and interleukin-6 (IL-6) [10, 11]. Inducing immunogenic necrosis of cancer cells can also activate anti-tumor immune response in vivo. ATP and HMGB1 are damage related substances of immunogenic cell death, which have been shown to be released during Ferroptosis and act as an immunogenic signal associated with the immunogenicity of iron-dead cancer cells [12]. Consequently, ferritinophagy exhibits distinct immune response characteristics. Moreover, ferritinophagy also has the characteristics of general autophagy, such as the altered expression content of autophagy-related genes autophagy related 5 (ATG5) and autophagy related 7 (ATG7) [13], increased LC3II/LC3I ratio [14] and genomic instability and mutations (Fig. 1b). It has been demonstrated that ferritinophagy in diffuse B-cell lymphoma cells triggered by eprenetapopt (APR-246) is characterized by tumor protein p53 (TP53) mutations [15]. Macroautophagy, endosomal microautophagy and ferritinophagy all also require the participation of Tax1 binding protein 1 (TAX1BP1) [16].

#### Ferritinophagy and ferroptosis

Ferritinophagy is the apoptotic pathway of ferroptosis, which has common characteristics with ferroptosis but different from ferroptosis (Table 1).

**Table 1:** Comparison of ferroptosis and iron ferritinophagy autophagy characteristics.

	ferritinophagy	References	ferroptosis	References
mode of cell death	selective autophagy induced apoptosis	[9]	iron-dependent programmed cell death	[17]
cellular morphology	autophagosome	[35]	cell swelling and plasma membrane rupture	[40]
autophagy receptor	NCOA4	[3]	HPCAL1	[27]
organelle characteristics	phagocytosis and digestion in lysozyme	[3]	Mitochondrial shrinkage, mitochondrial membrane density concentration, mitochondrial crest reduction or disappearance, mitochondrial membrane rupture	[21–25]
molecular marker	ATG5, ATG7, LC3II/LC3Iratio, TP53 mutation	[14–16]	SSBP1, PTGS2, SLC7A11, ACSL4, RGS4	[28]
immunological markers	DCN	[10, 11]	ATP, HMGB1	[12]
epigenetic regulation	miRNA, Methylation and acetylation	[29]	Histone ubiquitination, methylation, miRNA, and the immune microenvironment	[30–33]
cellular metabolism	protein degradation	[3]	Antioxidant system imbalance, amino acid metabolism disorder, nucleotide metabolism disorder	[26]

Ferroptosis is an iron-dependent form of programmed cell death characterized by the accumulation of LPOs [17], which is generated by the iron-dependent oxidation of polyunsaturated fatty acids [18]. The accumulation of LPOs in the cell membrane leads to membrane damage and ultimately, cell death. Ferroptosis is regulated by a variety of factors, including iron, glutathione peroxidase 4 (GPX4), and other antioxidant enzymes [19, 20]. Additionally, ferroptosis disrupts the equilibrium between redox reactions and promotes oxidative damage to cellular organelles. This oxidative damage manifests in morphological changes such as smaller mitochondria, reduced or absent mitochondrial cristae, and concentrated rupture of the outer mitochondrial membrane [21–25]. In addition to the imbalance of antioxidant system caused by excessive accumulation of LPOs, the disorder of amino acid metabolism and nucleotide metabolism is also related to ferroptosis [26]. In contrast to the autophagy receptor NCOA4, which is unique to ferritinophagy, a new ferroptosis receptor, Hippocalcin like 1, is also recently identified [27]. Compared to the ferroptosis specific molecular markers GPX4, prostaglandin-endoperoxidesynthase2, solute carrier family 7 member 11 (SLC7A11), acyl-CoA synthetase long-chain family member 4, regulator of G-protein signaling 4, single stranded DNA binding protein 1 (SSBP1) [28], it has been observed that ferritinophagy controls disturbed protein mechanisms encoding and regulating iron transcripts, including differences in miRNA, methylation and acetylation [29]. Histone ubiquitination, methylation, miRNA, and the immune microenvironment also modulate ferroptosis in cancer cells [30–33].

Although ferroptosis and ferritinophagy are distinct processes, they are both involved in the regulation of iron metabolism in cells. Ferroptosis can lead to the release of iron from cells, while ferritinophagy can increase the availability of iron for cellular use. The precise relationship between these two processes and their role in iron metabolism is an active area of research [34].

#### Ferritinophagy regulatory mechanism

Ferritinophagy is regulated by several mechanisms that control both synthesis and degradation of ferritin. One of the key regulatory mechanisms is the control of intracellular iron levels, which can trigger or inhibit the process of ferritinophagy in response to the changes in cellular iron homeostasis. First, ferritin binds to NCOA4 via the FTH1 subunit to form a complex. Next, the double walled proto-autophagosome membrane contains the ferritin-NCOA4 complex to form a completely closed structure. Finally, the autophagosomes transfer to the lysosomes and fuse with each other, and the ferritin degrades and releases iron in the lysosomes [35]. Among them, intracellular proteins involved in the regulation of iron homeostasis also affect the sensitivity of cells to ferritinophagy.

Ferritinophagy is dependent on the selective ferritin degradation of iron as it is sensitive to iron ions and the iron transport proteins expression. The intracellular iron atom has a complete iron regulatory network. Once the iron uptake increase, a decrease in iron export or stores due to autophagic degradation of ferritin can result in the development and progression of ferroptosis promoted [36, 37].

#### Regulation of ferritinophagy through NCOA4

**Fig. 2:**
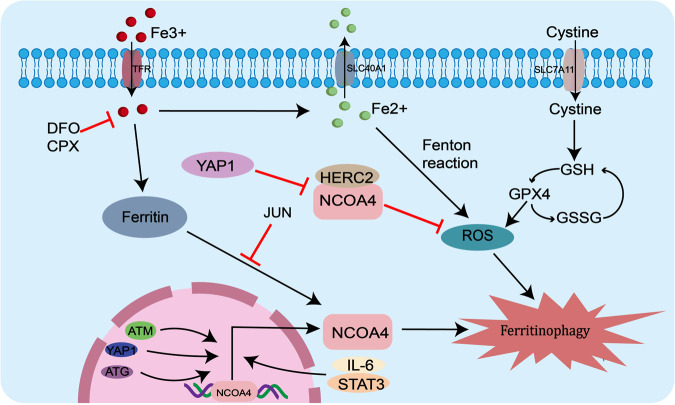
The ferritinophagy pathway map is regulated by NCOA4. Molecules such as ATG, ATM, YAP1 and signaling pathways such as JNK-JUN can regulate the occurrence of intracellular ferritinophagy by regulating the expression of NCOA4. YAP1 can also inhibit cell death by blocking the interaction of NCOA4 with FTH1 and reducing the production of reactive oxygen species.

Ferritin is a cellular iron storage complex that can undergo lysosomal degradation via the selective autophagic adapter NCOA4, releasing iron for cellular use. Current studies have shown that alterations in many molecules can regulate the occurrence of iron autophagy by modulating the expression of NCOA4. For example, autophagy-associated genes (ATG) can activate NCOA4, and overexpression of NCOA4 promotes intracellular iron ion transportation and cellular ferritinophagy, thereby inducing ferroptosis [38, 39]. While knockdown of NCOA4 or ATG significantly reverses the reduction of transferrin, attenuates iron overload and lipid peroxidation, and alleviates cellular ferroptosis [40]. DNA double-strand break-related genes ataxia telangiectasia mutated (ATM) is indispensable in ferroptosis, and ATM can promote NCOA4-ferritin interactions to maintain ferritinophagy by phosphorylating NCOA4 [41]. Not only can changes at the molecular level affect NCOA4 expression, but signaling pathways can also regulate NCOA4 expression. In cells, NCOA4 binds to FTH1 and then connects to LC3II in the lysosome, releasing ferrous iron and enhancing the Fenton reaction to promote ferritinophagy, thus inducing the onset of ferroptosis [10, 42, 43]. The interaction between NCOA4 and FTH1 can be blocked by yes-associated protein 1, a key regulator of the Hippo signaling pathway, who can down-regulates GPX4, FTH1 and SLC7A11, and up-regulates siderofexin (SFXN1) and NCOA4 to reduce ROS production to inhibit hepatocyte ferroptosis [44]. NCOA4 is also regulated by the IL-6/STAT3 signaling pathway, which protects cardiomyocytes from ferritinophagy mediated ferroptosis by inhibiting STAT3 [45]. SUN et al. demonstrated that NCOA4 is dependent on the regulation of the JNK-JUN signaling pathway where JUN can bind to the promoter of NCOA4 to inhibit the interaction between NCOA4 and ferritin, thereby increasing ferritin autophagic degradation and promoting ferroptosis in chondrocytes [46]. In addition, heat damage or ionizing radiation can also cause cellular damage through NCOA4-mediated ferritinophagy [47–50]. All of above suggest that NCOA4 expression is associated with ferritinophagy in cancer cells [51, 52] (Fig. 2).

### Regulation of ferritinophagy through the Nrf2/HO-1 signaling pathway

**Fig. 3:**
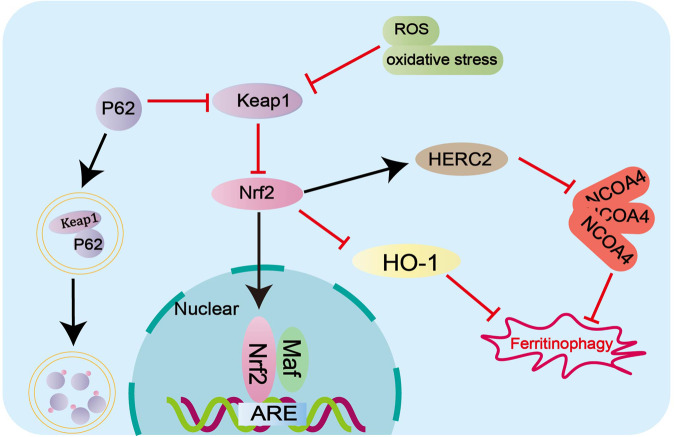
Nrf2 signaling pathway regulates ferritinophagy. Not only can Nrf2/HO-1 signaling pathway and p62-Keap1-Nrf2 pathway regulate ferritinophagy, Nrf2 also controls E3 ubiquitin protein ligase antibody (HERC2), mediates NCOA4 turnover through the ubiquitin-proteasome system, and inhibits the occurrence of ferritinophagy.

Heme oxygenase-1 (HO-1) is a rate-limiting enzyme in heme degradation that catalyzes the oxidative degradation of heme and the release of free iron, mediating cellular ferritinophagy and ferroptosis [53]. HO-1 is regulated by nuclear factor red lineage 2-related factor 2 (Nrf2), a key transcription factor that plays a protective role in ferroptosis. It has been demonstrated inhibiting the Nrf2/HO-1 signaling pathway is the feasibility of killing cancer cells [54, 55]. Also, Nrf2 controls the E3 ubiquitin protein ligase antibody (HERC2), which mediates NCOA4 turnover through the ubiquitin-proteasome system, reducing NCOA4 levels and resulting in blocked ferritinophagy [56]. Meanwhile, under conditions of oxidative stress, Nrf2 dissociates from Kelch-like ECH-associated protein 1 (Keap1), transfers Nrf2 to nucleus after accumulation and activates transcription of targeted anti-oxidative stress genes, thereby promoting cellular oxidative stress [57]. Moreover, Nrf2 can inhibit ferroptosis in colorectal cancer by binding with Keap1 to down-regulate Nrf2 [58]. It has been shown in previous studies that the p62-Keap1-Nrf2 pathway regulates protein conversion from cytoplasmic LC3, who is a characteristic protein in autophagy process, to membrane LC3II [59]. Therefore, Nrf2 has an effect on ferritinophagy-dependent ferroptosis through autophagy protein activation (Fig. 3).

### Other pathways modulate ferritinophagy

The regulation of ferritinophagy involves not only its characteristic markers, but also the coordination with other pathways. One such example is the association of the oncogene RAS with ferritinophagy, where its related KRAS-MAPK pathway enhances cell viability through the synthesis of mitochondrial sulfur-cluster proteins [60]. Additionally, the activation of ferritinophagy by the AMPK-mTOR-ULK1 axis is crucial for ferroptosis, as indicated by the increased levels of LC3 and MDA, and the decreased levels of FTH [61]. Moreover, the regulation of the SLC38A9-mTOR axis and cholesterol also play a role in the dependence of ferritinophagy [62]. A mutant of isocitrate dehydrogenase 1 (IDH1R132H) has been shown to enhance ferritinophagy in gliomas through the inhibition of the PRMT1-PTX3 axis signaling pathway [63]. Tripartite motif-containing protiens (TRIMs), a family of E3 ubiquitin ligases, play a crucial role in the degradation of endogenous proteins via the ubiquitin-proteasome system. Specifically, TRIM11 has been identified as an inhibitor of intracellular ferritinophagy by modulating NCOA4, ferritin, and Fe2+ levels through UBE2N/TAX1BP1 signaling [64]. Furthermore, circRNAs in non-coding RNAs have also been implicated in the regulation of ferritinophagy, exerting a positive regulatory effect on ferroptosis in hepatocellular carcinoma cells by suppressing ALKBH5-mediated autophagy inhibition [65]. Related studies have demonstrated that SLC7A11/GPX4 expression and ferritinophagy can not only direct the susceptibility to ferroptosis, respectively [62], but also the antioxidant SLC7A11/system xc-/xCT or GPX4 can over-activate ferritinophagy leading to ferroptosis [66, 67]. Therefore, GPX4 can regulate ferroptosis not only directly but also indirectly by regulating ferritinophagy [62, 68].

### Application of ferritinophagy in cancers

The process of cancer development involves alteration of many markers, and intracellular iron concentration and ferritin imbalance play an important role in ferritinophagy-mediated cancer cell growth. Ferritinophagy, an inducible pathway in the onset of ferroptosis, can induce ferroptosis by targeting ferritin, which provides a new strategy for cancer therapy. Dysfunction of the enzyme body autophagy pathway and impaired iron metabolism are associated with many diseases in humans [69]. Previous studies have confirmed that decreased iron transporter protein (FPN) and increased transferrin receptor (TFR1) are associated with prognosis in ovarian cancer [70]. In addition, TFR1 expression is also associated with prognosis in lung cancer [71], cervical cancer [72], and hepatocellular carcinoma [73]. A growing body of research evidence suggests the involvement of ferritinophagy in cancer evolution.

### Application of ferritinophagy in anti-cancer

#### Ferritinophagy promotes cell death and inhibit Epithelial-Mesenchymal Transition (EMT) by affecting the lipid peroxidation pathway

**Fig. 4:**
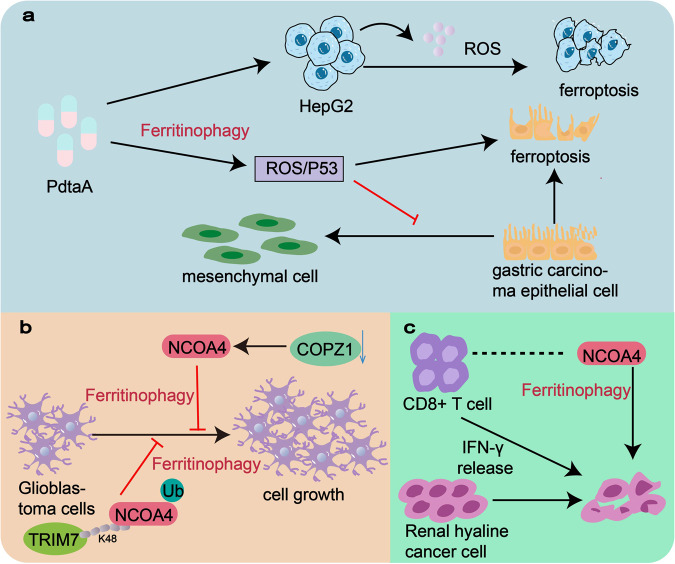
Application of ferritinophagy in anti-cancer. **a** Iron chelator 2-pyridylhydrazone dithiocarbamate s-acetate acid (PdtaA) promotes ROS production and induces ferroptosis in HepG2 cells through ferritinophagy. Moreover, it can inhibit epithelial-mesenchymal transformation (EMT) of gastric cancer cells through the ROS/p53 pathway mediated by ferritinophagy. **b** Knockdown of coatomer protein complex subunit zeta 1 (COPZ1) inhibited glioblastoma (GBM) cell proliferation by increasing NCOA4. The K48-linked chain in TRIM7 binds directly to NCOA4 and ubiquitinates it, inhibiting the growth of human glioblastoma cells mediated by NCOA4 by promoting ferritinophagy. **c** Low expression of NCOA4 gene in renal clear cell carcinoma is associated with reduced immune cell infiltration and impaired IFN-γ receptor signaling pathways. Moreover, CD8+ T cells can induce cell death to eliminate cancer by specifically enhancing lipid peroxidation of cancer cells.

Unrestricted cellular proliferation is a notable pathological characteristic observed in cancer. The process of ferritinophagy initiates lipid peroxidation, resulting in membrane impairment and cellular demise by degrading ferritin, all of which are closely linked to the progression of cancer. Genes or pathways that regulate the lipid peroxidation system have an impact on the growth and development of cancer cells [74, 75]. Genes or pathways that regulate the lipid peroxidation system have an impact on the growth and development of cancer cells. For instance, iron chelator 2-pyridylhydrazone dithiocarbamate s-acetate acid (PdtaA) has been found to enhance the production of reactive oxygen species (ROS) and induce cell cycle arrest in HepG2 cells through ferritinophagy [76]. Additionally, PdtaA down-regulates GPX4 and xCT, leading to ferroptosis. Another research team has demonstrated the chelator’s ability to inhibit epithelial-mesenchymal transition (EMT) in gastric cancer cells through ferritinophagy mediated the ROS/p53 pathway [77] (Fig. 4a). The resulting increased lipid peroxide production can promote autophagy-mediated ferroptosis in cancer cells.

#### Ferritinophagy inhibits cancer cell growth through the mediation of NCOA4

Cellular autophagic processes inhibit cancer cell activity mediated by ferritin. The main mode of iron released from ferritin is selective autophagy mediated by NCOA4, therefore, NCOA4-mediated ferritinophagy has a key role in cancer progression. For instance, the inhibition of coatomer protein complex subunit zeta 1 (COPZ1) leads to a decrease in glioblastoma (GBM) cell proliferation through the upregulation of NCOA4 and the facilitation of ferritin degradation [78]. TRIM7’s K48-linked chain directly binds to NCOA4 and ubiquitinates it, thereby impeding the growth of human GBM cells mediated by NCOA4 and promoting ferritinophagy [79] (Fig. 4b).

#### Ferritinophagy and immune response coordinate anti-cancer

The body is susceptible to interference by foreign pathogens when its immune function is reduced, and cancers are prone to occur at this time. Cancer cells that die from ferritinophagy release certain molecules to stimulate the immune system to fight against cancers, and enhanced ferritinophagy and immune activation can synergistically reinforce each other in inducing cancer cell death. NCOA4 not only facilitates cellular ferritinophagy but also plays a role in cancer cell-mediated immune responses. For instance, the diminished expression of NCOA4 in renal clear cell carcinoma has been found to govern unfavorable cancer prognosis and impaired infiltration of immune cells. In the present study, it was observed that CD8+ T cells activated through immunotherapy can effectively induce cancer cell death by augmenting lipid peroxidation in cancer cells [51] (Fig. 4c). Furthermore, NCOA4 can act as a target for macrophages to regulate iron overload and as a signal for inflammatory molecules [80].

#### Ferritinophagy in non-cancer

Currently, ferritinophagy primarily exhibits an anti-cancer effect in cancers, although there is evidence suggesting that the regulation of ferritinophagy-related pathways does not impact cell function. For example, the silencing of NCOA4 in colon cancer cells did not yield any noteworthy disparity in the expression of ferritin and TFR1, thereby suggesting that the growth of colon cancer cells does not necessitate the participation of ferritinophagy [81]. During the investigation into the process of lipid peroxide formation in ferritinophagy, a research team conducted experiments that revealed the ability of the ferroptosis inducer erastin to promote ferritinophagy in HeLa cells expressing NCOA4. Conversely, another ferroptosis inducer, RAS-selective lethal 3, was found to delay the death of HeLa cells overexpressing NCOA4 [82]. This demonstrates that whether ferritinophagy can regulate the onset of ferroptosis depended on the inducing compounds and the downstream pathways activated by cell death.

### Application of targeting ferritinophagy in cancer treatment

#### Chemotherapy exert anti-cancer effect by targeting ferritinophagy

Autophagy is an adaptive response to metabolic and therapeutic stress in a manner that can be applied to clinical therapeutic targets and prognostic monitoring. Regulation of any process in the ferritinophagy pathway can lead to cellular alterations. In the first place, NCOA4 plays a role in cancer therapy. Studies have shown that lipopolysaccharide (LPS) can regulate ferritin conversion by inhibiting NCOA4 as a therapeutic target for cancer [83]. Powdered tetrandrine citrate (TetC), a novel highly water-soluble powdered cycloheximide salt, has strong anticancer activity not only in chronic myeloid leukemia, but also in breast cancer cells by inhibiting GPX4 expression and activation of NCOA4 to promote ferritinophagy in cancer cells inducing ferrogenic cell death [80]. In addition, other pathways have been validated in ferritinophagy-mediated cancer therapy. For example, the process of iron transportation into mitochondria by lysosomes during ferritinophagy can serve as a potential target for enhancing pancreatic cancer survival rates and overcoming treatment resistance [84]. The reactive oxygen species (ROS) generated through ferritinophagy can activate the p53 and PHD2/HIF-1α signaling pathways, while also inhibiting the iron chelator 2,2’-di-pyridylketone hydrazone dithiocarbamate s-butyric acid (DpdtbA), thereby inducing EMT in gastric cancer cells [85]. The administration of the bromodomain protein BRD4 inhibitor (+)-JQ1 in cancer cells reliant on ferritinophagy triggers the ferroptosis process. Consequently, cancer cells treated with JQ1 exhibit an increased LC3II/LC3I ratio, reduced FTH1 expression, and decreased expression of autophagy-related genes ATG5 and ATG7 [86]. Above demonstrating that more and more ferritinophagy-related molecules are becoming targets in cancer therapy.

Therapeutic mechanisms for common anticancer drugs have also been found in the ferritinophagy pathway. Low-dose cisplatin combined with ursolic acid inhibits cancer cell growth by activating autophagic degradation of ferritin and overloading intracellular iron ions [87]. Heavy floor saponin formosaninC (FC) induces ROS formation and GPX4 damage, promoting ferroptosis and ferritinophagy in triple-negative breast cancer MDA-MB-231 cells and increasing their chemosensitivity to cisplatin [88]. The combination of artesunate and hepatocellular carcinoma advanced first-line drug sorafenib induces oxidative stress and ferritinophagy in hepatocellular carcinoma cells and promotes lysosome-dependent FTL degradation to improve the efficacy of single drug [89]. Clinically, we need to know not only the drug efficacy, but also the adverse effects and the conditions of application of each drug to avoid additional serious damage to patients body. Rifampicin, the most common causative agent of antituberculosis drug-induced liver injury (AT-DILI), can reduce the hepatotoxicity of rifampicin by activating the autophagic pathway to reduce ferritinophagy and ferroptosis [90]. Additionally, the anticancer antibiotic Adriamycin was observed to enhance ferritinophagy by affecting the SPATA2/CYLD pathway, leading to NCOA4 depletion and ferroptosis induction in cardiomyocytes [91]. Carboplatin, a first-line drug for the treatment of human retinoblastoma (RB), develops acquired multidrug resistance (MDR) after long-term treatment, and its resistant mechanism can be eliminated by inducing autophagy-dependent ferroptosis [92]. In addition, it has also been recognized that the same drug efficacy can be exerted through multiple pathways. Dihydroartemisinin, a derivative of artemisinin, has been shown to have cytotoxic effects on a variety of malignant cancer cells of human origin, which can induce ferritinophagy and lysosomal degradation resulting in cell death in an autophagy-independent manner [93].

#### Nanomaterials and photodynamic therapy acts anti-cancer effect by targeting ferritinophagy

Nanomaterials have emerged as a valuable tool in the field of cancer treatment, particularly through their ability to modulate the ferritinophagy pathway. For instance, MoS2 nanosheets have been found to induce ferroptosis by degrading ferritin within the lysosome and inhibiting the function of recombinant FPN via NCOA4-dependent [94]. Moreover, nanomaterial-based systems have also been shown to connect ferritinophagy with the anticancer effects of the immune system. In this context, Zinc oxide nanoparticles exhibit promising biomedical applications by leveraging microautophagy/autophagy to induce ferroptosis in human umbilical vein endothelial cells, leading to vascular inflammation and ferritinophagy [95].

In addition to common pharmacological treatments, photodynamic therapy (PDT) has established a new targeted therapeutic approach for osteosarcoma. PDT can promote ferroptosis in human osteosarcoma cells through NCOA4-mediated ferritinophagy and GPX4 inactivation, synergistic over-accumulation of lipid peroxides (LPOs), and significant induction of cytochrome c-activated mitochondrial apoptosis [96]. Immunity is closely related to cancers and activation of the immune system can also co-mediate cancer therapy with activation of ferritinophagy [97]. PDT-mediated activation of T cells and ferroptosis activation are connected, suggesting a theoretical basis for a new paradigm of cancer therapy [98]. Related studies have confirmed that increased ferroptosis can also promote the anticancer effects of immunotherapy. For instance, testosterone has been found to have a significant impact on prostate cancer, as supraphysiological levels of testosterone can impede the growth of prostate cells through ferritinophagy and nuclear autophagy. Additionally, it activates immune signaling pathways driven by nucleic acid sensors, leading to an increased migration of cytotoxic immune cells to the cancer site [99].

#### Immunity plays an anti-cancer role by targeting ferritinophagy

The human immune system has the role of immune surveillance, defense and regulation, which is closely related to the occurrence and development of cancers. When the immune function of body decreases, the cancer is easy to invade, so the cancer immunity can be used to study the occurrence, development and regression of cancers [100]. Furthermore, the search for cancer markers remains an ongoing and extensive endeavor. The emergence of new antigenic markers for cancer cells is a prominent immunological feature of cancer cells [101], and the release of inflammatory factors by the immune system stimulated by ferritinophagy death cells [7] provides us with good direction. Immune checkpoint blockade is a powerful oncologi inflammatory factors treatment modality for a wide range of human malignancies [102], and targeting the cancer ferritinophagy pathway might be an immune checkpoint blockade therapy. Preclinical data suggest that immune checkpoint blockade can act synergistically with radiotherapy through agonism of innate immune sensing pathways activated by DNA damage [103]. Notably, two separate studies have concurrently demonstrated that immunotherapy-activated CD8+ T cells augment lipid peroxidation in ferroptosis cancer cells. Furthermore, it has been observed that heightened ferroptosis contributes to the effectiveness of immunotherapy in combating cancer [104, 105]. In addition, the reduction in iron and FTH due to ferroptosis inhibition impedes dendritic cell-mediated anticancer immunity and therefore may interfere with immunotherapy [106, 107]. How the induction of ferroptosis through ferritinophagy modulates immune efficacy is still unknown, and the mechanisms controlling the immune response to specific ferritinophagy remain poorly defined. A current challenge in oncology is to effectively integrate immunotherapy with conventional therapies, involved radiotherapy. Recent years have provided insights into the molecular mechanisms [108], pharmacological regulation [109] and functional significance of ferritinophagy in health and disease [110]. However, the mediators of its immune response elicitation remain poorly defined. Immunogenic cell death is an important factor in the success of anticancer therapy [111]. Although it is promising that the application of the ferritinophagy pathway to therapy, how ferritinophagy works with the immune system to fight cancer cell processes remains unclear.

## Conclusion

Great progress has been made in the discovery of ferritinophagy research. Many studies have shown that ferritinophagy is closely related to the occurrence and development of cancer. Therefore, exploring the characteristics and regulatory mechanisms of ferritinophagy will help us to better understand its role in cancer. In the previous sections we have learned that ferroptosis is often accompanied by the occurrence of cell swelling and plasma membrane rupture, whereas apoptotic cells often exhibit cell shrinkage and blistering of the plasma membrane. The autophagic degradation pathway usually protects cells from apoptosis, but selective ferritinophagy promotes iron cell apoptosis [40], which enlightens us whether ferritinophagy has different features from ferroptosis in terms of cell morphological changes.

At present, there are teams that have linked iron autophagy with clinical drugs to anti-cancer in vitro, which greatly accelerates the application of iron autophagy in preclinical. Currently, targeted therapy is a widely used therapeutic approach, but still generates resistance to drug-induced autophagy and death in cancer cells [112]. Ferritinophagy, the death of non-apoptotic cells, is an emerging cancer therapeutic target, which has the potential to resist the therapeutic effect of cancer therapy without apoptosis [113]. It still has very important clinical significance to target ferritinophagy against cancer as there are no clinically relevant drugs for cancer treatment, but the application of nanomaterials in iron autophagy brings us new hope in the treatment of cancer. However, a lot of efforts are needed to move from preclinical research to clinical application. We should not only explore the application of single drugs in cancers, but also urgently search for combination drugs to improve the efficacy of drug therapy. Exploring the synergistic effect of immunotherapy and biotherapy combined in ferritinophagy provides us with a good way forward.
